# Ancient Chinese Methods Are Remarkably Effective for the Preparation of Artemisinin-Rich Extracts of Qing Hao with Potent Antimalarial Activity

**DOI:** 10.3390/molecules15020804

**Published:** 2010-02-04

**Authors:** Colin W. Wright, Peter A. Linley, Reto Brun, Sergio Wittlin, Elisabeth Hsu

**Affiliations:** 1 Bradford School of Pharmacy, University of Bradford, West Yorkshire, BD7 1DP, UK; E-Mail: p.a.linley@bradford.ac.uk (P.A.L.); 2 Swiss Tropical Institute, Socinstrasse 57, PO Box, CH-4002, Basel, Switzerland; E-Mails: Reto.Brun@unibas.ch (R.B.); Sergio.Wittlin@unibas.ch (S.W.); 3 Institute of Social and Cultural Anthropology, University of Oxford, 51 Banbury Road, Oxford, OX2 6PE, UK; E-Mail: Elisabeth.hsu@anthro.ox.ac.uk (E.H.)

**Keywords:** chinese herbal texts, *Artemisia annua*, artemisinin, malaria

## Abstract

Ancient Chinese herbal texts as far back as the 4th Century *Zhou hou bei ji fang* describe methods for the use of Qing Hao (*Artemisia annua*) for the treatment of intermittent fevers. Today, the *A. annua* constituent artemisinin is an important antimalarial drug and the herb itself is being grown and used locally for malaria treatment although this practice is controversial. Here we show that the ancient Chinese methods that involved either soaking, (followed by wringing) or pounding, (followed by squeezing) the fresh herb are more effective in producing artemisinin-rich extracts than the usual current method of preparing herbal teas from the dried herb. The concentrations of artemisinin in the extracts was up to 20-fold higher than that in a herbal tea prepared from the dried herb, but the amount of total artemisinin extracted by the Chinese methods was much less than that removed in the herbal tea. While both extracts exhibited potent *in vitro* activities against *Plasmodium falciparum*, only the pounded juice contained sufficient artemisinin to suppress parasitaemia in *P. berghei* infected mice. The implications of these results are discussed in the context of malaria treatment using *A. annua* infusions.

## 1. Introduction

In the early 1970s, Chinese scientists screening traditional Chinese medicinal herbs in the search for new antimalarials isolated artemisinin from the herb known as Qing Hao (generally identified as *Artemisia annua* L. but may have originally referred to *A. apiacea*) [1]. Artemisinin is an unusual sesquiterpene lactone incorporating an endoperoxide group. It has potent antiplasmodial activity and was shown in clinical trials to be highly effective against malaria, including patients with cerebral malaria and patients with malaria parasites resistant to chloroquine [2]. In contrast to other antimalarials such as quinine, (used for treating chloroquine-resistant malaria), artemisinin was found to be remarkably non-toxic and the importance of its discovery against the background of a rising incidence of multidrug-resistant malaria parasites cannot be underestimated. Several semi-synthetic artemisinin derivatives including artemether, arteether and sodium artesunate are now in clinical use worldwide for the treatment of malaria [3]. However, antimalarial drugs such as the artemisinin derivatives are expensive and are not accessible to the majority of people who are at risk of malaria, especially in Africa with the result that approximately 1 million people, mostly children, die from malaria each year [4]. 

The desperate need for affordable medicines for the treatment of malaria has stimulated the search for alternative treatments and in particular the use of *A. annua* as a locally grown herbal treatment for malaria [5]. For this purpose the dried herb is usually used in the form of an infusion prepared by adding boiling water to the herb, allowing to stand and then drinking the strained liquor. In China, Qing Hao was documented more than 1,600 years ago for the treatment of ‘intermittent fevers’ by the famous physician Ge Hong (284-363 A.D.) in the *Zhou hou bei ji fang* which may be translated as ‘Emergency prescriptions kept up one’s sleeve’. Interestingly, in this text specific instructions are given: ‘Qing Hao, one bunch, take two sheng (2 × 0.2 L) of water for soaking it, wring it out, take the juice, ingest it in its entirety [6]. Other methods in later references involve soaking the plant in urine rather than water and pounding the fresh herb to produce a juice [7]. In view of these interesting instructions, here we report experiments designed to simulate the methods described in the ancient Chinese texts and evaluate their potential to provide locally accessible malaria treatment.

## 2. Results and Discussion 

### 2.1. Extraction of artemisinin by traditional methods

*A. annua* herb was harvested from 4-month old high-artemisinin yielding plants (Anamed) grown in the UK. Leaves and small stems were soaked in water and briefly drained before ‘wringing by hand’ and collecting the juice expressed. No juice could be obtained from the herb by wringing before soaking in water. The ‘wrung juice’ was kept separate from the water used to soak the herb and the artemisinin concentration of each was determined using gas-liquid chromatography. The results (Table 1) showed that the ‘wrung juice’ method was surprisingly effective in extracting artemisinin from fresh *A. annua* herb. Herb soaked in water (sufficient to cover the herb), for 12 h before wringing yielded wrung juice containing more artemisinin (72.6 mg/L) than that soaked for only 2 h (45.9 mg/L). The artemisinin concentrations achieved were nearly five and three times higher, respectively, than that achieved by preparing an infusion from dried herb prepared from the same plants (14.5 mg/L). The solubility of artemisinin in water is poor on account of its non-polar nature and a concentration of only 10.6 mg/L was reported when boiling water was added to pure artemisinin [8]. In the plant, artemisinin is stored in 10-celled biseriate glandular trichomes present on the epidermis of the aerial parts [9]. The effectiveness of the ‘wrung’ method may be due, in part, to ‘mechanical’ effects resulting in the crushing and/or removal of the glandular trichomes. Another factor may be the presence of essential oil in the glandular trichomes that is composed of a mixture of monoterpenes and sesquiterpenes [10] and these may possibly aid solubilisation. Herb soaked for 12 h before wringing as compared to 2 h produced wrung juice containing almost 50% more artemisinin, an effect that may be related to the absorption of water by the herb during soaking, perhaps resulting in swollen and more fragile glandular trichomes. It is possible that 12 h is not the optimal time for soaking and further experiments to determine this would be worthwhile. Unfortunately, the Chinese texts do not give any details in this respect. Although the ‘wrung’ method provides a product with greater artemisinin concentration, the efficiency of the process in terms of the proportion of the total amount of artemisinin present in the plant material is relatively poor (4.51 and 7.27% for the 2 h and 12 h ‘wrung’ juice respectively) compared to the dried leaf infusion (53.8%). However, in addition to the artemisinin present in the ‘wrung’ juice, an amount is also present in the water in which the herb was soaked (6.55 and 13.2 mg/L for the 2 h and 12 h samples respectively) resulting in total efficiencies of 9.08 and 14.1% respectively. It is likely that traditionally, the liquid remaining after soaking as well as the wrung juice would have been consumed, thus obtaining maximum benefit from the preparation. 

Pounded juice (prepared by pounding the herb with a mortar and pestle followed by squeezing the resulting pulp by hand) was found to have the highest artemisinin concentration (293 mg/L), and this was more than 20-fold that of the dried herb infusion and 4-fold higher than that in the 12 h wrung juice. Again, the extraction efficiency was relatively low (14.9%) but it is likely that this could be improved considerably by mechanically squeezing the pulp after pounding as with hand-squeezing the yield of juice was only 0.18 mL/g fresh herb. However, it was not possible to produce juice by mechanically crushing the non-pounded herb in a mangle. The ‘pounding’ method thus provides a product with a remarkably high artemisinin content compared to the other methods but the effort required to produce it is high by comparison and as the resulting juice is dark green and viscous it may not be as palatable as the almost colourless ‘wrung’ juice. 

These results clearly demonstrate that the wrung juice and the pounded juice methods described in ancient Chinese medical texts are effective in extracting artemisinin from the herb. However, the results described here were obtained using a selected variety of *A. annua* (Anamed) with a relatively high artemisinin content (0.033% in the fresh herb) that is not representative of wild-type *A. annua*. In order to compare the latter with a non-selected variety, wrung juice (2 h) was prepared from fresh herb containing 0.009% artemisinin (Chiltern Seeds). This juice contained 24.6 mg/L artemisinin which, although half that of the 2 h Anamed plant wrung juice is similar to that of the Anamed dried leaf infusion (Table 1). The dried herb (Chiltern Seeds) was found to contain 0.072% artemisinin (data not shown) which is within the range of 0.02–1.07% reported in the literature for non-hybrid samples grown in different parts of the world [11] while the dried herb from the Anamed plants contained 0.273%. The above results show that, as might be expected, herb containing higher amounts of artemisinin yields juices with higher artemisinin content although the efficiency of extraction with the lower-yielding (Chiltern Seeds) herb was slightly higher (6.24%) for the 2 h wrung juice compared to that for the 2 h Anamed wrung juice (4.51%). Despite the lower artemisinin content of the Chiltern herb, the artemisinin content of the 2 h wrung juice was comparable to the Anamed dried herb infusion clearly demonstrating that the ancient Chinese method of soaking and then hand wringing the herb has the potential to yield juice with a surprisingly high artemisinin content even from plants containing a modest content of artemisinin that occur in the wild. In this study, 12 h wrung juice or pounded juice from the Chiltern herb were not prepared but by analogy with the Anamed herb, these may be expected to yield juices containing higher artemisinin levels compared to the 2 h wrung juice. It should also be noted that young (4 month old) plants were used for this study but maximum artemisinin yields are obtained at or near the flowering stage so that there may be potential for even higher yields of artemisinin. 

### 2.2. Antimalarial activity

*In vitro* evaluation of the wrung and pounded juices prepared from the Anamed plants against *P. falciparum* [12], showed that the pounded leaf juice was the most potent as a 1:500,000 dilution of the juice inhibited parasite growth by 50% while the 2 h and 12 h wrung juices were about half as potent (Table 1). In each case, the antiplasmodial activities correlated well with the artemisinin content. However, the IC_50_ values in terms of artemisinin content were 6–18x lower compared to that for the control artemisinin (IC_50_ = 3.8 ng/mL) indicating that the activity of the juices could not be entirely accounted for by their artemisinin content. The pounded, 2 h and 12 h wrung juices from the Anamed herb and the 2 h wrung juice from the Chiltern herb were tested orally in mice infected with *Plasmodium berghei* [12]. Only the pounded juice was found to suppress parasitaemia in the mice (Table 2). One, two and three volumes of 500 μL (each with an artemisinin content corresponding to a dose of 9 mg/kg) given at 12 h intervals (to avoid administering excessive volumes) were found to suppress parasitaemia by 52, 95, and 96% respectively, compared to untreated infected control mice. The two and three-volume doses used corresponding to 18 and 27 mg/kg artemisinin respectively resulted in a suppression of parasitaemia, (95 and 96% respectively) that was comparable to that seen with a control dose of pure artemisinin (30 mg/kg single oral dose) which suppressed parasitaemia by 88%. Interestingly, the suppression of parasitaemia seen with the two-volume dose (18 mg/kg), was comparable to the suppression seen with a single dose of 30 mg/kg artemisinin. This effect is higher than expected and may be due to giving the treatment in two divided doses 12 h apart as artemisinin has a short elimination half life. Another possibility is that other constituents of *A. annua* have contributed to the antimalarial efficacy. Treatment of infected mice with the wrung juices had no effect on parasitaemia, (data not shown) but this is explicable in terms of their artemisinin concentrations and the dose volume limitation of 3 × 500 μL - the artemisinin content of 1.5 mL of the 12 h wrung juice is equivalent to less than 5 mg/kg bodyweight. The results of both the *in vivo* experiments as well as the *in vitro* antiplasmodial assay are consistent with the artemisinin contents of the juices. 

### 2.3. Implications for malaria treatment

Currently, the treatment of malaria with infusions made from the dried herb of locally grown *A. annua* especially using high-artemisinin yielding varieties is being promoted by some organisations. This is a very attractive option where people do not have access to, or cannot afford, effective drugs for the treatment of malaria caused by *P. falciparum*. Clinical evidence regarding the efficacy of *A. annua* infusions is limited to a few small studies carried out in adult patients with uncomplicated malaria [13]. The results suggest that symptoms and parasitaemia are rapidly cleared and it has been shown that plasma levels of artemisinin well in excess of that considered necessary to inhibit the growth of *P. falciparum in vitro* (10 ng/mL) are achieved [14]. On the other hand, in studies where patients were followed up [15,16], high rates of recrudescence occurred because not all of the parasites had been killed; this may be related to the short half-life of artemisinin and to other factors that are not fully understood [17]. Paradoxically, although artemisinin is a highly effective antimalarial, it is difficult to achieve a high cure rate (i.e. without recurrence of the infection due to recrudescence), with daily doses of 500 mg, far higher than the amount provided by infusions. Another important limitation of the studies is that they were carried out in adult patients who were likely to have some immunity to malaria so that the results cannot be extrapolated to children and non-immune adults who are the groups most at risk of severe disease and death due to malaria. A further problem is the concern that the use of *A. annua* infusions may encourage the development of malaria parasites resistant to artemisinin. There is evidence that artemisinin-based antimalarials are becoming less effective [18] and the World Health Organisation has called for a halt to the marketing and sale of malaria medicines that contain only artemisinin or its derivatives – a second antimalarial drug must be given in order to prevent recrudescence and minimise the risk of resistance development [19]. 

It is often argued that the use of herbal teas is not likely to lead to the development of malaria parasite resistance because other plant constituents such as flavonoids may act synergistically with artemisinin. The antiplasmodial action of artemisinin has been reported to be enhanced by the methoxylated flavones artemetin and casticin which are constituents of *A. annua* but this has only been observed *in vitro* [20]. In the clinical studies cited above, recrudescence was a major problem suggesting that even if artemisinin does act synergistically with other plant constituents, this interaction is insufficient to prevent this phenomenon. 

## 3. Experimental

### 3.1. Plant material and sample preparation

A high-artemisinin yielding cultivar of *A. annua* (seeds obtained from Anamed International, Schafweide 77, 71364 Winnenden, Germany) together with an unspecified variety for comparison (Chiltern Seeds, Ulverston, UK) were grown at Hailey House, Oxfordshire UK from seed germinated in an unheated greenhouse and seedlings were transplanted outdoors when they were large enough to handle. Four months-old plants of each variety were harvested and used for experiments on the same day. Leaves and small stems (150 g) from five plants of each were soaked separately in beakers containing sufficient water to cover the herb for 2 or 12 h, removed, allowed to drain briefly and then wrung by hand. The juices expressed were each extracted three times with hexane and then the combined hexane extract was dried over anhydrous sodium sulphate and concentrated using a rotary evaporator at 40 °C. Dried extracts were stored at −20 °C until analysed using gas-liquid chromatography. Pounded juice was prepared by ‘bruising’ the fresh herb using a mortar and pestle until a ‘pulp’ was produced; the juice was squeezed out by hand and then extracted three times with hexane as above. Samples of the fresh and dried herbs were similarly extracted with hexane in order to determine their total artemisinin contents. 

### 3.2. Artemisinin analysis and determination of antiplasmodial activity

The artemisinin content of the hexane extracts was determined using a Perkin Elmer 8600 or Clarus 400 gas liquid chromatograph (Perkin Elmer, UK) using a previously reported method [21]. A minimum of 3 separate analyses was carried out for each sample and the mean value determined. Samples of wrung and pounded juice were assessed for *in vitro* activities against *Plasmodium falciparum* (NF54) using the method described previously [12]. Samples of the wrung and pounded juice were also tested orally in mice infected with *P. berghei* [12]. These tests were commenced within 24–48 h of preparing the extracts in order to minimize possible loss of artemisinin.

## 4. Conclusions

This study has shown that following the instructions for the use of Qing Hao given in ancient Chinese medical texts produces extracts that have markedly higher artemisinin concentrations than the current method of preparing aqueous infusions. For the treatment of a 50 Kg adult patient with malaria, it has been estimated that a daily dose of 100 mg artemisinin would be required [22]. Using the pounded juice described above would require the patient to consume 0.34 L per day whereas ~7 L of the dried herb infusion would be needed to provide the same dose of artemisinin. Adopting these methods for the preparation of herbal medicines from *A. annua* herb for the treatment of malaria could result in higher artemisinin concentrations. However, unfortunately, this is not likely to reduce the rate of recrudescence seen when artemisinin or *A. annua* teas are used for malaria treatment. In order for malaria treatment with *A. annua* herbal preparations to be effective and acceptable, ways need to be found to eliminate the problem of recrudescence and also to minimise the potential for the development of artemisinin-resistant parasites. This could possibly be achieved by combining Qing Hao with other herbs. Clinical studies are also needed to show whether severe disease can be treated and deaths prevented, especially in children and other non-immune malaria patients. For the time being, due to the reasons discussed above, malaria treatment with preparations made with *A. annua* herb cannot be recommended. Instead, we recommend that further research on herbal *A. annua* treatments be undertaken that address these problems, as the ethical dilemma remains acute for those seeking to help malaria victims who do not have access to effective antimalarial drugs.

## Figures and Tables

**Table 1:** Artemisinin content, efficiency of artemisinin extraction and *in vitro* antiplasmodial activities of preparations made from *A. annua* herb grown from Anamed (high artemisinin) and Chiltern (low artemisinin) seeds.

Type of *A. annua* (artemisinin % w/w)^a^	Method^b^	Total artemisinin in sample;	Amount of artemisinin extracted, volume of juice and artemisinin concentration in juice	Proportion of total artemisinin extracted (Extraction efficiency), (%)	*In vitro* activity vs. *P. falciparum* as IC_50_ (Dilution of juice giving IC_50_ and artemisinin concentration at this dilution)
Anamed, fresh herb (artemisinin 0.033%, n = 5)	Wrung (2 h)	49.5 mg in 150g	2.23 mg, 48.6 mL, ≡ 45.9 mg/L	4.51	1:222,222 ≡ 0.21 ng/mL artemisinin
as above	Wrung (12 h)	as above	3.60 mg, 49.3 mL, ≡ 72.6 mg/L	7.27	1:222,222 ≡ 0.33 ng/mL artemisinin
Anamed, dried herb (artemisinin 0.27%, n = 4)	Dried herb infusion (10 g/L)	27 mg in 10 g	-, -, 14.5 mg /L	53.8	NT
Anamed, fresh herb (artemisinin 0.033%, n = 5)	Pounded	43.2 mg in 131 g	6.44 mg, 22.0 mL, ≡ 293 mg/L	14.9	1:500,000 ≡ 0.59 ng/mL artemisinin
Chiltern, fresh herb (artemisinin 0.009%, n = 4)	Wrung (2 h)	13.5 mg in 150 g	0.843 mg, 34.3 mL, ≡ 24.6 mg/L	6.24	1:48.780 ≡ 0.50 ng/mL artemisinin
Artemisinin					3.8 ng/mL

^a^ Artemisinin content of herb samples was determined by extracting with hexane several times and analysing the extract using gas-liquid chromatography; values obtained were taken to represent 100%. ^b^ Wrung juices were prepared by soaking 150 g fresh herb in sufficient water to cover the herb for either 2 or 12 h; after removing the herb excess water was allowed to drain before wringing out the juice by hand. Pounded juice was prepared by pounding fresh herb in a mortar and then squeezing the resulting pulp by hand; the yield of juice was 0.18 mL/g. The infusion was prepared by adding 1000 mL boiling water to the dried herb and allowing to stand until cool; 10 g dried herb is equivalent to 34.3 g fresh herb. ^c^ Artemisinin content of extracts was determined by extracting several times with hexane and analysing the extracts as for the herb samples above. NT, not tested.

**Table 2:** *In vivo* evaluation of orally administered fresh pounded juice (Anamed) against *P. berghei* in mice.

Extract/drug	Dose	Parasitaemia (%)	Suppression of Parasitaemia (%)
Pounded juice (Anamed)	500 μL ^a^ × 1	16	52
	500 μL × 2	1.6	95
	500 μL × 3	1.4	96
Artemisinin	30 mg/kg × 1	3.9	88
Untreated control		34	0

^a^ 500 μL correspond to doses of 9 mg/kg artemisinin.
